# Supplementary material for: HIVprotI: an integrated web based platform for prediction and design of HIV proteins inhibitors
Source: J Cheminform. 2018 Mar 9;10:12. doi: 10.1186/s13321-018-0266-y (PMC5845081; doi:10.1186/s13321-018-0266-y)
Supplement: Supplementary file 2 — Additional file 2. Source code of HIVProtI web server. [file 13321_2018_266_MOESM2_ESM.zip › HIVprotI_Source-code/hivprotI/weka-3-6-6/doc/weka/filters/supervised/instance/package-summary.html]

weka.filters.supervised.instance


---


|  |  |  |
| --- | --- | --- |
| **Overview** \| **Package** \| Class \| **Tree** \| **Deprecated** \| **Index** \| **Help** \| **Weka's home** | |  |
| **PREV PACKAGE**   **NEXT PACKAGE** | **FRAMES**    **NO FRAMES**     **All Classes** |  |


---

## Package weka.filters.supervised.instance

| **Class Summary** | |
| --- | --- |
| **Resample** | Produces a random subsample of a dataset using either sampling with replacement or without replacement.  The original dataset must fit entirely in memory. |
| **SMOTE** | Resamples a dataset by applying the Synthetic Minority Oversampling TEchnique (SMOTE). |
| **SpreadSubsample** | Produces a random subsample of a dataset. |
| **StratifiedRemoveFolds** | This filter takes a dataset and outputs a specified fold for cross validation. |

---


|  |  |  |
| --- | --- | --- |
| **Overview** \| **Package** \| Class \| **Tree** \| **Deprecated** \| **Index** \| **Help** \| **Weka's home** | |  |
| **PREV PACKAGE**   **NEXT PACKAGE** | **FRAMES**    **NO FRAMES**     **All Classes** |  |


---
